# Efficacy and safety of ipilimumab in elderly patients with pretreated advanced melanoma treated at Italian centres through the expanded access programme

**DOI:** 10.1186/1756-9966-33-30

**Published:** 2014-04-04

**Authors:** Vanna Chiarion Sileni, Jacopo Pigozzo, Paolo Antonio Ascierto, Antonio Maria Grimaldi, Michele Maio, Lorenza Di Guardo, Paolo Marchetti, Francesco de Rosa, Carmen Nuzzo, Alessandro Testori, Emilia Cocorocchio, Maria Grazia Bernengo, Michele Guida, Riccardo Marconcini, Barbara Merelli, Giorgio Parmiani, Gaetana Rinaldi, Massimo Aglietta, Marco Grosso, Paola Queirolo

**Affiliations:** 1 Melanoma Cancer Unit, Oncology Institute of Veneto IRCCS, Via Gattamelata, 64, 35128 Padua, Italy; 2 Unit of Melanoma, Cancer Immunotherapy and Innovative Therapy, Istituto Nazionale Tumori Fondazione “G. Pascale”, Naples, Italy; 3 Medical Oncology and Immunotherapy Division, University Hospital of Siena, Istituto Toscano Tumori, Siena, Italy; 4 Medical Oncology, National Cancer Institute, Milan, Italy; 5 Medical Oncology, Dermopathic Institute of the Immaculate IDI-IRCCS, Rome, Italy; 6 Medical Oncology, Sant'Andrea Hospital, University Sapienza, Rome, Italy; 7 Immunotherapy Unit, Romagna National Cancer Institute, Meldola, Italy; 8 Department of Medical Oncology A, Regina Elena National Cancer Institute, Rome, Italy; 9 Divisione melanoma e sarcomi muscolo-cutanei, Istituto Europeo di Oncologia, Milan, Italy; 10 Melanoma Medical Oncology Division, Istituto Europeo di Oncologia, Milan, Italy; 11 University Hospital St John the Baptist, Turin, Italy; 12 Medical Oncology Department, National Cancer Research Centre, “Giovanni Paolo II”, Bari, Italy; 13 University Hospital Pisa, “Gathered Hospitals of Santa Chiara”, Pisa, Italy; 14 Department of Oncology and Hematology, Unit of Clinical and Translational Research, “Papa Giovanni XXIII” Hospital, Bergamo, Italy; 15 Unit of Immuno-Biotherapy of Melanoma, San Raffaele Hospital, Milan, Italy; 16 “Paolo Giaccone” Polyclinic University Hospital, Palermo, Italy; 17 Institute of Cancer Research and Treatment, Piedmont Oncology Foundation, Candiolo, Italy; 18 University of Torino, Turin, Italy; 19 Medical Oncology A, National Institute for Cancer Research, Genoa, Italy

**Keywords:** Melanoma, Ipilimumab, Expanded access, Elderly patients, Treatment outcome, Safety

## Abstract

**Background:**

Elderly patients with metastatic melanoma have different disease characteristics and a poorer prognosis than younger patients. Data from clinical trials and expanded access programmes (EAPs) suggest ipilimumab confers a consistent survival benefit and has a similar safety profile across different age groups of patients with metastatic melanoma. Here we report the efficacy and safety of ipilimumab 3 mg/kg in elderly patients enrolled in an EAP in Italy.

**Methods:**

Patients aged > 70 years with pretreated melanoma received ipilimumab 3 mg/kg every 3 weeks for four doses through an EAP. Tumour response was evaluated at baseline and after completion of induction therapy using immune-related response criteria and patients were monitored throughout the treatment period for adverse events (AEs), including immune-related AEs.

**Results:**

The immune-related disease control rate among 188 evaluable patients was 38%, including four patients with an immune-related complete response, 24 with an immune-related partial response and 44 with immune-related stable disease. Median progression-free survival (PFS) was 4.0 months and the 1- and 2-year PFS rates were 21% and 12%, respectively. Median overall survival (OS) was 8.9 months; 1- and 2-year OS rates were 38% and 22%, respectively. The safety profile of ipilimumab was consistent with that observed in the general population of the Italian EAP and treatment-related AEs generally resolved within a median of 2 weeks with treatment as per protocol-specific guidelines.

**Conclusions:**

These results suggest ipilimumab is a feasible treatment option in elderly patients with metastatic melanoma. Ipilimumab treatment was generally well tolerated and resulted in clinical benefit and extended survival in elderly patients treated at centres in Italy.

## Background

Historically, patients with unresectable Stage III or Stage IV (advanced) melanoma had limited treatment options and poor survival outcomes, with older patients having a particularly dismal prognosis [1,2]. In 2010, there were an estimated 13.6 melanoma-related deaths per 100 000 US inhabitants aged > 65 years compared with 1.2 per 100 000 US inhabitants aged ≤ 65 years [3]. Current epidemiological data suggest the incidence of melanoma continues to rise in the elderly population despite indications that it has plateaued in younger people [3,4]. Combined with a rapid increase in the proportion of elderly people, this has resulted in melanoma becoming an increasingly important health concern in the developed world [5].

A number of explanations for the poor prognosis of elderly patients with melanoma have been proposed. Older melanoma patients may be more predisposed to distant metastasis arising from the haematological distribution of tumour cells than younger patients due to changes in lymphatic drainage with ageing [6]. In addition, elderly patients present with thicker melanomas, a higher mitotic rate and increased incidence of ulceration [7], all of which are associated with a worse prognosis [1]. It is likely, however, that the high mortality rates among elderly patients result from a number of age-related variables preventing optimal management of this disease [8].

One confounding factor that may contribute to the poor prognosis of elderly patients with metastatic melanoma is a weakening of the immune system with age, a process referred to as immunosenescence. Therefore, the possibility of using immune-based therapies to promote immune function is an attractive therapeutic option [8,9]. In 2011, the novel immunotherapy agent ipilimumab was the first agent approved for the treatment of patients with advanced melanoma in over three decades [10]. Ipilimumab is a fully human monoclonal antibody directed against cytotoxic T-lymphocyte-associated antigen-4 (CTLA-4), a negative regulator of T-cell-mediated immune responses. By blocking CTLA-4, ipilimumab enables prolonged T-cell activation, proliferation and tumour infiltration, thereby potentiating endogenous antitumour responses [11].

Ipilimumab 3 mg/kg is now approved in over 40 countries for the treatment of adult patients with advanced melanoma. In Phase III trials, ipilimumab treatment significantly extended overall survival (OS) compared with control in both pretreated and treatment-naϊve patients [12,13], and follow-up data from clinical trials suggest ipilimumab can provide durable clinical benefit and long-term survival [13-15]. Furthermore, retrospective analyses of clinical trial data suggest the survival benefit conferred by ipilimumab is independent of age, performance status and stage of metastasis, despite the identification of these variables as significant prognostic indicators [1,16,17].

Expanded access programmes (EAPs) provide an opportunity to assess the efficacy and safety of ipilimumab at its approved dose of 3 mg/kg in elderly patients outside of a clinical trial, in a setting more representative of daily practice. Efficacy and safety results from the Spanish and US EAPs suggest ipilimumab 3 mg/kg is a feasible treatment option in elderly patients with metastatic melanoma [18-20]. Here, we describe the efficacy and safety of ipilimumab 3 mg/kg in elderly (> 70 years old) patients with metastatic melanoma treated at Italian centres participating in the European EAP. Data from other patient subgroups treated in the Italian EAP have been published previously [21,22].

## Methods

### Patients

Patients were eligible to be included in the EAP if they had life-threatening unresectable Stage III or Stage IV melanoma and had failed to respond or were intolerant to at least one prior systemic treatment. Ipilimumab was available on physicians’ request where no alternative treatment option was available. An Eastern Cooperative Oncology Group (ECOG) performance status of 0, 1 or 2 was required, and an interval of at least 28 days since completion of treatment with chemotherapy, biochemotherapy, surgery, radiation, or immunotherapy recommended. The protocol for the EAP was approved by a local independent ethics committee and all participating patients provided signed informed consent before enrolment. The study was approved by the ECs of all participating centers.

### Treatment and clinical assessment

Ipilimumab 3 mg/kg was administered intravenously over 90 minutes, every 3 weeks for four doses. Disease evaluation was performed at baseline and after completion of induction therapy using immune-related response criteria (irRC) [23]. Clinical response was defined as immune-related complete response (irCR), partial response (irPR), stable disease (irSD) or progressive disease. Immune-related disease control (irDC) was defined as an irCR, irPR or irSD lasting ≥ 3 months. All patients were monitored for safety throughout the EAP, and adverse events (AEs), including immune-related AEs (irAEs), graded according to the Common Terminology Criteria for Adverse Events, version 3.0.

### Statistical analysis

Patient and disease characteristics were analysed using descriptive statistics with data expressed as relative frequencies (percentages) for discrete variables, or median and range for continuous variables. Progression-free survival (PFS) and OS were estimated using Kaplan–Meier analysis and expressed as median values with corresponding two-sided 95% confidence intervals (CIs).

## Results

### Patients

A total of 855 patients participated in the EAP from June 2010 to January 2012 across 55 Italian centres, including 193 patients (23%) aged > 70 years (median age, 75; range 71–88 years) of which 27 were aged ≥ 80 years. Baseline patient and disease characteristics are shown in Table 1. Of the 193 elderly patients, 132 patients (68%) received all four doses, 24 (12%) received three doses, 17 (9%) received two doses and 20 patients (10%) received one dose of ipilimumab 3 mg/kg. Reasons for not completing all four doses of ipilimumab therapy comprised disease progression (*n* = 22), death (*n* = 18), deterioration without progression (*n* = 3), AEs unrelated to treatment (*n* = 4), dose skipping (*n* = 2), patient refusal (*n* =1), loss to follow up (*n* = 1), and unknown reasons (*n* = 3). Only 7 patients (4%) discontinued for reasons of treatment-related toxicity.

**Table 1:** Baseline patient characteristics

**Characteristic (*N*** **= 855)**	**Patients aged > 70 years**	**Patients aged ≤ 70 years**
Total number of patients	193	662
Median age, years (range)	75 (71–88)	55 (16–70)
Male/female, *n* (%)	112 (58)/81 (42)	348 (53)/314 (47)
ECOG performance status, *n* (%)		
0	105 (54)	458 (69)
1	83 (43)	184 (28)
2	5 (3)	20 (3)
Time from diagnosis, months (range)	35 (3–280)	40 (3–280)
LDH level, *n*/*n* (%)^a^		
< 1.10 ULN	108/175 (62)	336/545 (62)
≥ 1.10 ULN	67/175 (38)	209/545 (38)
Number of previous therapies, *n* (%)		
1	128 (66)	369 (56)
2	41 (21)	192 (29)
≥ 3	24 (13)	101 (15)
Previous therapy, *n* (%)		
Dacarbazine	113 (59)	377 (57)
Fotemustine	54 (28)	268 (41)
Platinum-based chemotherapy	42 (22)	274 (41)
Temozolomide	40 (21)	149 (23)
Interferon	22 (11)	172 (26)
BRAF inhibitor	8 (4)	51 (8)
Patients with brain metastases, *n* (%)	17 (9)	129 (20)
Patients with liver metastases, *n* (%)	75 (39)	264 (40)

^a^LDH data unavailable for 135 patients.

ECOG, Eastern Cooperative Oncology Group; LDH, lactate dehydrogenase; ULN, upper limit of normal.

### Efficacy

#### Tumour assessment

With a median follow-up of 7.9 months (mean 9.7 months; range 1–31 months), the irDC rate (irDCR) among 188 evaluable patients aged > 70 years was 38% (Table 2). This included four patients (2%) with an irCR, 24 (13%) with an irPR and 44 (23%) with irSD at any time according to irRC, for an immune-related best overall response rate (irBORR) of 15%. Five elderly patients were not evaluable for response due to toxicity (*n* = 1), loss to follow up (*n* = 1), only receiving one dose of ipilimumab (*n* = 1) or unknown reasons (*n* = 2). The median duration of irDC in elderly patients was 11.5 months (95% CI 9.3–13.7). The irDCR among 26 evaluable patients aged ≥ 80 years was 31%, comprising one patient (4%) with an irPR and seven patients (27%) with irSD. With a median follow-up of 6.7 months (range 1–34), the irDCR among 645 evaluable patients aged ≤ 70 years was 33%. Of these, 25 patients (4%) had an irCR, 58 (9%) an irPR and 131 (20%) had irSD at any time according to irRC. The irBORR in patients aged ≤ 70 years was therefore 13%.

**Table 2:** Tumour response

	**Patients, *n *** **(%)**		
**Response according to irRC**	**Aged > 70 years (*n*** **= 188)**	**Aged ≥ 80 years (*n*** **= 26)**	**Aged ≤ 70 years (*n*** **= 645)**
irCR	4 (2)	0 (0)	25 (4)
irPR	24 (13)	1 (4)	58 (9)
irSD	44 (23)	7 (27)	131 (20)
irPD	116 (62)	18 (69)	431 (67)
irBORR	28 (15)	1 (4)	83 (13)
irDCR	72 (38)	8 (31)	214 (33)

[Note to authors: summarised these data as a table and added data for patients ≥80 years].

irBORR, immune-related best overall response rate; irCR, immune-related complete response; irDCR, immune-related disease control rate; irPD, immune-related progressive disease; irPR, immune-related partial response; irRC, immune-related response criteria; irSD, immune-related stable disease.

#### Survival

**Figure 1:**
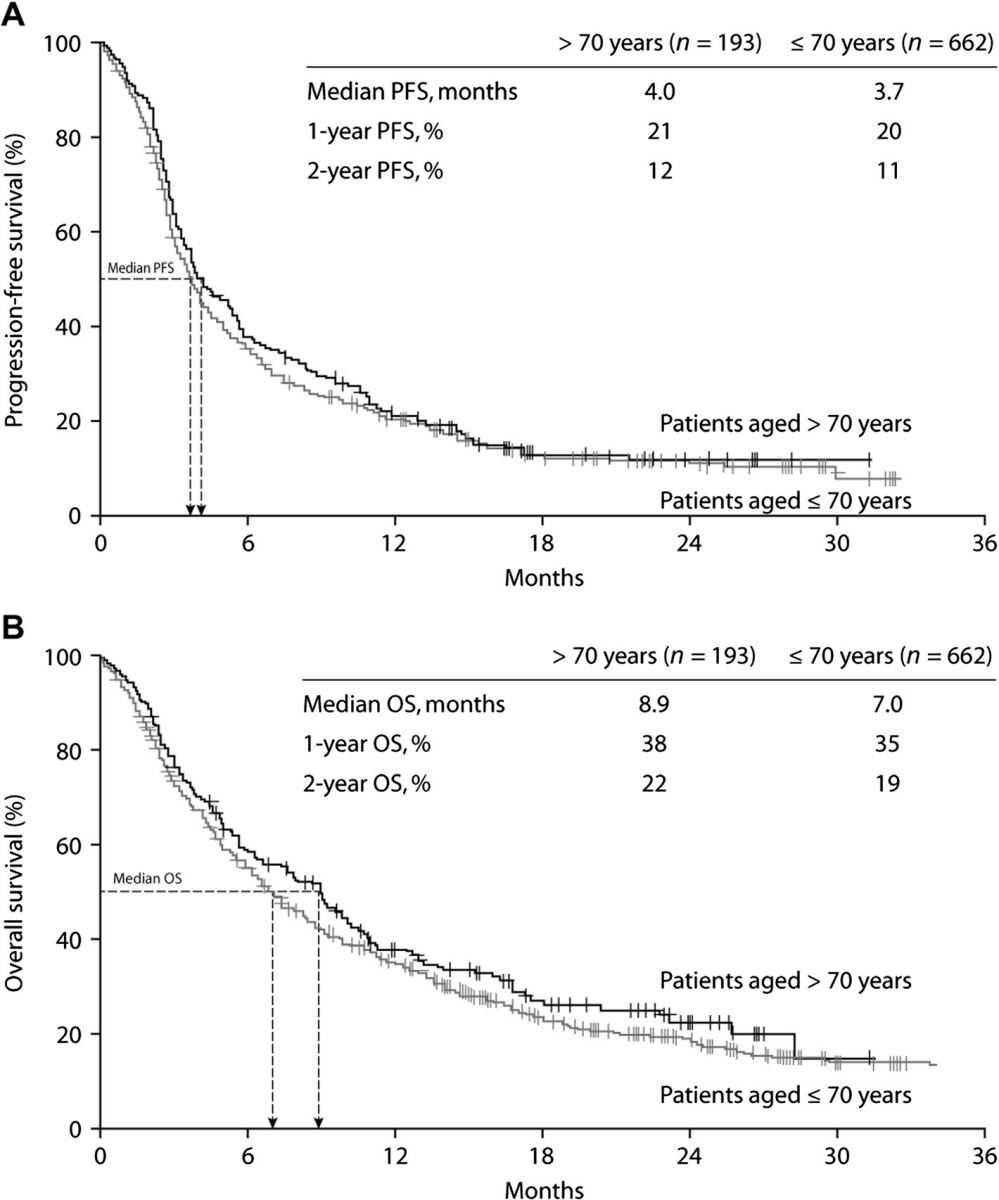
**Kaplan-Meier estimates of progression free survival and overall survival by patient ages. A**. Progression-free survival. **B**. OS, overall survival; PFS, progression-free survival.

As of April 2013, median PFS in patients > 70 years old was 4.0 months (95% CI 3.0–5.0; Figure 1A); 1- and 2-year PFS rates were 21% and 12%, respectively. By comparison, median PFS in younger patients (≤ 70 years) was 3.7 months (95% CI 3.4–4.0), with 1- and 2-year PFS rates of 20% and 11%, respectively. In the elderly patient group (> 70 years old), median OS was 8.9 months (95% CI 7.2–10.6; Figure 1B); 1- and 2-year OS rates were 38% and 22%, respectively. For patients aged ≤ 70 years, median OS was 7.0 months (95% CI 6.1–7.9); 1- and 2-year OS rates in the younger age group were 35% and 19%, respectively. Differences between age groups in median PFS and median OS were not statistically significant (*P* = 0.33 and *P* = 0.17, respectively).

### Safety

The safety profile of ipilimumab in elderly patients was comparable to that in the wider EAP population [24]. Of the 193 patients aged > 70 years treated with ipilimumab, 96 (50%) reported an AE of any grade and among these 96 patients, 69 (36%) had AEs that were considered to be treatment-related. Respective numbers for the 662 patients aged ≤ 70 years were 303 (46%) and 217 (33%). The most frequently reported treatment-related AEs among patients aged > 70 years were pruritus, rash, diarrhoea, nausea and liver toxicity (experienced by at least 2% of patients; Table 3). Median time to onset of treatment-related AEs of any grade was 3 weeks (range 0.1–12 weeks). Grade III–IV AEs were reported by 19 patients (10%) and considered ipilimumab-related in 11 patients (6%). Median time to onset of treatment-related Grade III–IV AEs was 6 weeks (range 3–10 weeks). AEs were generally reversible with treatment as per protocol-specific guidelines. Median time to resolution of treatment-related AEs of any grade was 2.0 weeks (range 0.1–4), compared with a median of 1.7 weeks (0.1–11.1) among all patients treated in the EAP in Italy [24].

**Table 3:** Treatment-related AEs experienced by at least 2% of patients aged > 70 or ≤ 70 years

	**Patients aged > 70 years (*n*** **= 193), *n *** **(%)**		**Patients aged ≤ 70 years (*n*** **= 662), *n *** **(%)**	
**Treatment-related AEs experienced by at least 2% of patients**	**Any grade**	**Grade III–IV**	**Any grade**	**Grade III–IV**
Pruritus	11 (6)	0	47 (7)	1 (<1)
Rash	19 (10)	1 (<1)	45 (7)	3 (<1)
Diarrhoea	9 (5)	2 (1)	51 (8)	17 (3)
Nausea	5 (3)	0	42 (6)	2 (<1)
Liver toxicity	3 (2)	2 (1)	16 (2)	13 (2)

AEs, adverse events.

## Discussion

Elderly patients with metastatic melanoma have higher rates of overall and disease-specific mortality than younger patients [7]. Furthermore, older patients are more likely to have existing comorbidities, which often result in their exclusion from clinical trials of investigative new therapies [25]. The EAP in Italy provided the opportunity to assess the efficacy and safety of ipilimumab 3 mg/kg in elderly patients with advanced melanoma outside of a clinical trial setting.

Most other subgroup analyses have used a cut-off age of 65 years when reporting the use of ipilimumab in elderly patients [12,19,20,26]. Our results suggest ipilimumab treatment is equally effective and safe in patients with advanced melanoma who are aged over or under 70 years. This higher cut-off age may be more relevant to the challenges associated with cancer treatment in an aging society. Indeed, the cut-off for many clinical cancer studies is now 70 years and this is expected to be revised upwards so that 75 years may soon be the standard upper age limit for inclusion in a clinical trial [27,28]. Among the 855 patients who participated in the EAP in Italy, almost one quarter were aged > 70 years and were eligible for treatment. This figure is consistent with the proportion of patients > 70 years diagnosed with melanoma in Italy as recorded in the Italian cancer registry, demonstrating that the elderly patients treated as part of the EAP can be considered as representative of the general population of patients > 70 years with melanoma.

Elderly patients had long-lasting clinical responses and prolonged survival with ipilimumab 3 mg/kg. The irBORR and irDCR in patients aged > 70 years were similar to those observed in the wider population of the Italian EAP [24] and in 30 elderly patients (≥ 70 years old) treated at Spanish centres through the EAP [20]. One- and 2-year survival rates of 38% and 22% are also comparable with those reported for the total population and consistent with results from the US EAP, in which 1-year survival rates for patients < 65 years or ≥ 65 years were 38% and 37%, respectively [18]. In the Italian EAP, PFS and OS survival curves were comparable between older and younger patients. Although there was a tendency for survival to be better in the elderly patient cohort, the differences in median PFS and median OS between older and younger patients were not statistically significant and were most likely to chance since the inclusion and exclusion criteria were the same for all patients, as was the follow-up duration, This finding is also consistent with prespecified subgroup analyses of data from the Phase III trial of ipilimumab in pretreated patients, in which the survival benefit with ipilimumab monotherapy compared with gp100 monotherapy was slightly but not significantly greater in patients aged ≥65 years than in younger patients (<65 years) [12,16]. Similarly, in the registrational trial of vemurafenib, an inhibitor of mutated BRAF, no differences in survival or response were reported between older (≥ 65 years) and younger patients (< 65 years) with metastatic melanoma [29].

Ipilimumab is associated with irAEs, which may reflect the proposed mechanism of action [11,30]. Most irAEs are mild or moderate and, provided they are recognised early, can be resolved effectively with appropriate management [31]. Among patients > 70 years treated in the Italian EAP, ipilimumab was generally well tolerated with only 6% of patients experiencing Grade III–IV treatment-related AEs. In addition, most elderly patients received all four doses or discontinued treatment for reasons other than toxicity. The AE profile of ipilimumab in patients aged > 70 years was again consistent with that observed in the overall EAP population, with a similar incidence of Grade III–IV treatment-related AEs and no unexpected toxicities. The results were also in line with subgroup analyses of safety data from patients treated with ipilimumab in clinical trials, EAPs or as standard of care [12,19,24]. In the US EAP, 11% patients aged ≥ 65 years had a Grade III–IV irAE compared with 7% patients aged < 65 years [19]. Similarly, only four elderly patients (13%) treated in the Spanish EAP had a Grade III–V AE and no patients discontinued treatment due to toxicity [20]. Taken together, these results suggest that increased age does not compromise the tolerability of ipilimumab treatment. However, this requires further validation in very elderly patients, as recent data suggest that patients aged ≥ 75 years treated with vemurafenib are more likely to experience AEs than younger patients, including secondary skin lesions, decreased appetite and cardiac disorders [32].

The results of this EAP are particularly relevant as they show that ipilimumab provides a consistent survival benefit in patients aged over or under 70 years, despite the fact that the immune system often becomes less active in elderly people. Indeed, immunosenescence is an important risk factor for melanoma and is thought to affect all components of the immune system [8,9]. With regard to adaptive immunity, an age-related reduction in the proportion of naïve T cells occurs due to impaired T-cell development in the thymus. Functional defects in T-cell activity are also observed, partly due to a loss in costimulatory molecules, including CD28 [33]. However, ipilimumab may be particularly appropriate for the treatment of elderly patients because the expression of coinhibitory receptors such as CTLA-4 increases with age [34]. There is therefore a strong rationale for using anti-CTLA-4 therapy to treat elderly patients with metastatic melanoma in order to enhance adaptive immunity against this disease.

Most data regarding the use of ipilimumab in older patients are provided by EAP analyses. The EAPs are a valuable source of information regarding the efficacy and safety of ipilimumab outside of clinical trials, but they are also subject to limitations due to their retrospective, nonrandomised nature and the specific data collected. For example, the effect of patient comorbidities on the efficacy and safety of ipilimumab in elderly patients treated in the Italian EAP could not be assessed, as only limited comorbidity data were collected as part of the programme. In addition, it was not possible to stratify patients by activities of daily living (ADL) and instrumental ADL scales, which would have better characterised the patient population. However, these preliminary results suggest that ipilimumab is a safe and effective treatment option for elderly patients with metastatic melanoma. Continued follow-up in this patient population will provide long-term efficacy and safety results.

## Conclusions

Results from this analysis of elderly patients with advanced melanoma treated as part of an EAP in Italy suggest that ipilimumab 3 mg/kg is a well-tolerated treatment option, providing clinical benefit and extending survival in these patients. In addition, the clinical activity and safety profiles of ipilimumab in patients aged > 70 years were consistent with those observed in the wider population of the EAP. Although this analysis is subject to limitations, these results suggest that age should not be a deciding factor when considering whether to use ipilimumab to treat patients with advanced melanoma.

## Abbreviations

AE: Adverse event; CI: Confidence interval; CTLA-4: Cytotoxic T-lymphocyte-associated antigen-4; EAP: Expanded access programme; ECOG: Eastern Cooperative Oncology Group; irAE: Immune-related adverse event; irBORR: Immune-related best overall response rate; irCR: Immune-related complete response; irDC: Immune-related disease control; irDCR: Immune-related disease control rate; irRC: Immune-related response criteria; PFS: Progression-free survival; OS: Overall survival.

## Competing interests

Vanna Chiarion Sileni has received travel expenses for medical meetings and conferences and honoraria for advisory boards and consultancy from Bristol-Myers Squibb, GlaxoSmithKline, Merck Sharp & Dohme and Roche-Genentech. Paolo Ascierto has served in a consultancy/advisory role for Bristol-Myers Squibb, Merck Sharp & Dohme, Roche-Genentech, GlaxoSmithKline, Amgen and Celgene; he has also received research funding from Bristol-Myers Squibb, and honoraria from Bristol-Myers Squibb, Merck Sharp & Dohme, Roche-Genentech and GlaxoSmithKline. Michele Maio has had an advisory role and received funding for communication programs from Bristol-Myers Squibb, Roche-Genentech and Merck Sharp & Dohme and has received research funding from Bristol-Myers Squibb. Paolo Marchetti has had advisory roles for Bristol-Myers Squibb, GlaxoSmithKine and Novartis. Alessandro Testori has received honoraria and travel reimbursement for advisory boards from Bristol-Myers Squibb. Paola Queirolo has served in a consultant or advisory role for Bristol-Myers Squibb, GlaxoSmithKline and Roche-Genentech. All remaining authors have declared no conflicts of interest.

## Authors’ contributions

All authors made substantial contributions to the acquisition and interpretation of data, were involved in drafting the article or revising it critically for important intellectual content and provided final approval of the version to be published.
